# Metabolome and transcriptome integration reveals cerebral cortical metabolic profiles in rats with subarachnoid hemorrhage

**DOI:** 10.3389/fnagi.2024.1424312

**Published:** 2024-08-21

**Authors:** Haoran Lu, Teng Xie, Shanshan Wei, Yanhua Wang, Huibing Li, Baochang Luo, Xiaohong Qin, Xizhi Liu, Zilong Zhao, Zhibiao Chen, Rui Ding

**Affiliations:** ^1^Department of Neurosurgery, Renmin Hospital of Wuhan University, Wuhan, China; ^2^Department of Neurosurgery, Hanchuan Renmin Hospital, Hanchuan, China; ^3^Department of Oncology, Wuchang Hospital Affiliated to Wuhan University of Science and Technology, Wuhan, China

**Keywords:** subarachnoid hemorrhage, transcriptome, metabolome, secondary brain injury, inflammation

## Abstract

Subarachnoid hemorrhage (SAH) is a severe subtype of hemorrhagic stroke. The molecular mechanisms of its secondary brain damage remain obscure. To investigate the alterations in gene and metabolite levels following SAH, we construct the transcriptome and metabolome profiles of the rat cerebral cortex post-SAH using whole transcriptome sequencing and untargeted metabolomics assays. Transcriptomic analysis indicated that there were 982 differentially expressed genes (DEGs) and 540 differentially expressed metabolites (DEMs) between the sham group and SAH 1d, and 292 DEGs and 254 DEMs between SAH 1d and SAH 7d. Most notably, DEGs were predominantly involved in the activation of immune and inflammatory pathways, particularly the Complement and coagulation cascades, TNF signaling pathway, and NOD-like receptor signaling pathway. Metabolic analysis revealed that the metabolic pathways of Arginine and proline, Arachidonic acid, Folate biosynthesis, Pyrimidine, and Cysteine and methionine were remarkably affected after SAH. Metabolites of the above pathways are closely associated not only with immune inflammation but also with oxidative stress, endothelial cell damage, and blood–brain barrier disruption. This study provides new insights into the underlying pathologic mechanisms of secondary brain injury after SAH and further characterization of these aberrant signals could enable their application as potential therapeutic targets for SAH.

## 1 Introduction

Subarachnoid hemorrhage (SAH) is a severe subtype of stroke caused mainly by ruptured aneurysms. The disease is characterized by high morbidity and mortality and is increasingly more common in young adults. Survivors, often face a myriad of long-term challenges, including issues related to physical health, cognitive function, and socio-psychological well-being (Claassen and Park, 2022; Bandyopadhyay et al., 2024). A sudden, severe headache is one of the typical symptoms of SAH, and a head CT scan is the routine initial diagnostic method. If the CT scan results do not definitively diagnose, lumbar puncture becomes the next step for further diagnosis. The treatment for aneurysms mainly focuses on preventing blood flow into the aneurysm, with common treatment methods including aneurysm clipping surgery and endovascular coiling. SAH can lead to a variety of secondary complications, including early brain injury, increased intracranial pressure (ICP), and delayed cerebral ischemia (DCI). Potential mechanisms behind these complications encompass the activation of immune and inflammatory responses, microvascular dysfunction, abnormalities in brain metabolism, hematological changes, and cortical spreading depolarization (Helbok et al., 2011; Frontera et al., 2017; Keep et al., 2018; Eriksen et al., 2019; Gris et al., 2019). The pathophysiological processes involved in SAH are extremely complex, involving numerous and often overlapping factors, and our understanding of them remains quite limited. In-depth research into these complex pathological mechanisms and the identification of effective treatment targets are crucial for improving the long-term prognosis of SAH patients.

The interaction between genes and metabolites is both close and complex, forming the core components of many disease progressions. Utilizing multi-omics technologies to delve into these intricate biological processes allows us to gain a more precise understanding of the origins and progression pathways of diseases (Heo et al., 2021). The integration of transcriptomics and metabolomics is becoming an increasingly focused area of research. This interdisciplinary approach aids in identifying key genes, metabolites, and their related pathways, thereby uncovering new regulatory mechanisms and disease-related biomarkers (Jiang et al., 2022). For instance, metabolomics and transcriptomics combined analysis reveals that *Acanthopanax senticosus* can target the regulation of purine and lipid metabolic pathways in rats to treat ischemia–reperfusion injury (Chen et al., 2021). However, in SAH, the above-combined analysis has been rarely reported. Hence, by utilizing untargeted metabolomics analysis and RNA sequencing technology, we analyzed the interactions between the metabolome and transcriptome, aiming to explore the connections between genes and metabolites related to secondary brain injury after SAH. This approach helps us to gain a more comprehensive understanding of the pathophysiological mechanisms following SAH and provides novel insights into the treatment of this disease.

## 2 Materials and methods

### 2.1 Animals

In this study, adult Sprague Dawley rats (SD, aged 8–10 weeks, weighing approximately 260–280 grams) were sourced from Hunan Slike Jingda Laboratory Animal Co., Ltd. All rats were housed under standard environmental conditions with a 12-h light/dark cycle and had free access to water and food provided under conditions at the Experimental Center of Wuhan University People’s Hospital for 3 days. Throughout the experiment, we maintained each rat’s weight between 280 and 300 grams. All animal experimental protocols received formal approval from the Institutional Animal Care and Use Committee of Wuhan University People’s Hospital. In the experiment, 18 rats were randomly divided into three groups: sham group (*n* = 6), SAH 1 day group (*n* = 6), and SAH 7 day group (*n* = 6).

### 2.2 Establishment of animal models

This study established a rat SAH model through the intravascular perforation technique. Initially, rats were anesthetized via intraperitoneal injection of pentobarbital (dose of 40 mg/kg), then fixed on a heating platform for neck disinfection and skin preparation. Sterile surgical tools were used to incise and separate the neck skin and muscles, exposing the external carotid artery (ECA), internal carotid artery (ICA), and their branches. The distal end of the ECA was ligated and cut, and then the common carotid artery and ICA were temporarily closed with an arterial clip. Subsequently, a blunt-ended 4–0 nylon monofilament was inserted into the ICA through a small incision, the arterial clip was released, and the filament was slowly advanced towards the skull until resistance was felt, indicating the filament had reached the junction of the anterior cerebral artery and the middle cerebral artery. The filament was then further advanced 4–5 mm to perforate the artery and left in place for 10 s before being slowly withdrawn, thus inducing SAH. Afterward, the neck incision was disinfected and sutured, and the rats were allowed to recover on a warming blanket until the end of the surgery. Rats in the sham surgery group underwent the same surgical procedure, but the perforation step was not performed.

### 2.3 Sample preparation

At predetermined time points, samples of the entire cerebral cortex from the model side of the experimental rats were collected. Initially, these samples were rinsed with 0.9% saline to remove blood and connective tissue, followed by drying off surface liquid. Representative photos of the bottom of the rat brain in different groups are shown in Supplementary Figure S1. Subsequently, the samples were rapidly frozen in liquid nitrogen and stored in sterile preservation tubes at −80°C for subsequent transcriptomic and metabolomic analyses.

### 2.4 RNA extraction and library construction and quality assessment

We utilized Trizol reagent (Takara Biomedical Technology, Beijing, China) to extract total RNA from cerebral cortex samples and assessed the integrity and purity of RNA using NanoDrop 2000 (Thermo Fisher Scientific, Wilmington, DE, United States). Approximately 1 μg of total RNA per sample was used to construct, purify, and pool the cDNA libraries. These libraries were then sequenced on the Illumina HiSeq2500 platform (Bemec Biotechnology Co., Ltd., Wuhan, China). The entire RNA-Seq analysis process was managed by Biomarker Technologies (Beijing). Raw data (in fastq format) were processed to remove adapter-containing reads and low-quality reads based on Q20 (base error rate ≤ 1%) to ensure clean and high-quality data. The screening of differentially expressed genes (DEGs) was performed on the BMKCloud platform,^1^ using an adjusted *p*-value for false discovery rate (FDR), with *p* < 0.05 and |log2(Fold Change)| ≥ 1 as thresholds. GO functional enrichment and KEGG pathway analyses were completed on the OmicShare platform (Trott and Olson, 2010).^2^

### 2.5 Non-targeted metabolomics analysis

First, weigh 50 mg of the sample and add 1,000 μL of extraction solution containing an internal standard (methanol:acetonitrile:water = 2:2:1, internal standard (2-chloro-L-phenylalanine) concentration = 20 mg/L). Then, centrifuge at 4°C at 12000 rpm for 15 min and collect the supernatant for analysis. This experiment utilizes an LC/MS system composed of the Waters Acquity I-Class PLUS ultra-high performance liquid chromatograph paired with the Waters Xevo G2-XS QTof high-resolution mass spectrometer. In the analysis, the mobile phase A under positive ion mode is a 0.1% formic acid aqueous solution, and the mobile phase B is a 0.1% formic acid acetonitrile solution; for negative ion mode, the mobile phase A is a 0.1% formic acid aqueous solution, and the mobile phase B is a 0.1% formic acid acetonitrile solution. ESI ion source parameters are set as capillary voltage 2000 V (positive ion mode) or −1500 V (negative ion mode), cone voltage 30 V, ion source temperature 150°C, desolvation gas temperature 500°C, backflush gas flow rate 50 L/h, desolvation gas flow rate 800 L/h. Subsequently, raw data are processed for peak detection, extraction, comparison, and integration, and analyzed against mass spectrometry databases. Compound classification and pathway information for identified compounds are retrieved from the KEGG, HMDB, and LipidMaps databases. Principal Component Analysis (PCA) and Spearman correlation analysis are used to evaluate the consistency of samples within a group and quality control samples. Significant differences between compounds are calculated using the T-test (*p*-value). The OPLS-DA model is applied, and its robustness is verified through 200 permutation tests using the R package rolls, while multiple cross-validations are used to determine the importance of model variables projection (VIP). Differential metabolites are selected based on the OPLS-DA model’s fold change, p-value, and VIP value, with selection criteria being *p* < 0.05 and VIP > 1. Finally, the hypergeometric distribution test is used for enrichment analysis of differential metabolites in KEGG pathways, utilizing MetaboAnalyst (https://www.metaboanalyst.ca/, version 5.0) to identify related metabolic pathways.

### 2.6 Joint analysis of transcriptomics and metabolomics

In this study, we integrate transcriptomics and metabolomics data to perform joint pathway analysis through MetaboAnalyst5.0, aiming to obtain enriched maps of metabolic pathways. Additionally, the STRING database and the Cytoscape plugin CytoHubba are used to predict protein–protein interaction (PPI) networks and the top 15 central hub genes are identified. Furthermore, Spearman’s correlation analysis is employed to explore the relationships between the selected genes and metabolites.

### 2.7 Statistical analysis

All data are presented as mean ± standard error of the mean (SEM). Statistical analyses are performed using GraphPad Prism 9.0 (GraphPad Software, United States). Student’s *t*-test (two-tailed) or two-way analysis of variance (ANOVA) is appropriately used for comparisons between different groups. *p*-value <0.05 is considered statistically significant.

## 3 Results

### 3.1 Metabolomics analysis

**Figure 1:**
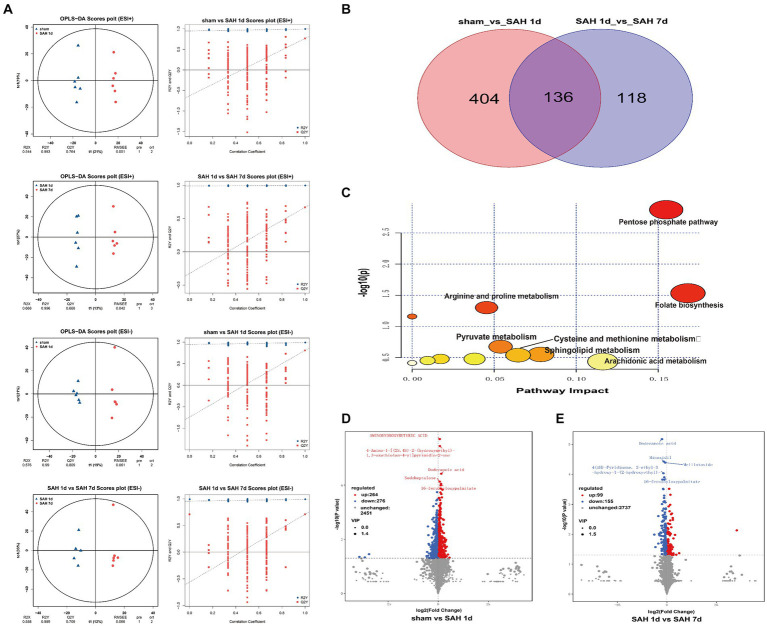
Metabolic characteristics of the sham surgery group, SAH 1d group, and SAH 7d group. **(A)** OPLS-DA and permutation score maps of the sham surgery group, SAH 1d group, and SAH 7d group in positive and negative ion mode. **(B)** Venn diagram of DMs. **(C)** Enrichment pathway diagram of differential metabolites. Volcano plots of differential metabolites in the sham surgery group and SAH 1d group **(D)**, SAH 1d group, and SAH 7d group. **(E)** Red indicates an upward adjustment; Green indicates a downward adjustment.

In this study, a non-targeted metabolomics approach based on LC–MS/MS is employed, identifying a total of 2,991 metabolites across both positive and negative ion modes. The samples exhibit good Spearman rank correlation (Supplementary Figures 2A,B). PCA score plots reveal overlaps between different groups (Supplementary Figures 2C,D). However, it’s important to note that PCA, as an unsupervised learning method, is primarily utilized to reveal the intrinsic distribution characteristics of the data. The OPLS-DA model is further analyzed, showing a clear separation effect between different groups. The results of the OPLS-DA indicate that the R^2^ is close to 1 and Q^2^ > 0.6, suggesting the model is stable and reliable. Through 200 iterations of permutation testing, the results indicate that the R^2^ value is greater than Q^2^, and the intercept of Q^2^ with the y-axis is less than 0. These results hint that the OPLS-DA model is neither overfitting nor underfitting, demonstrating stability and good predictive capability, effectively explaining the differences between the two groups of samples (Figure 1A).

**Figure 2:**
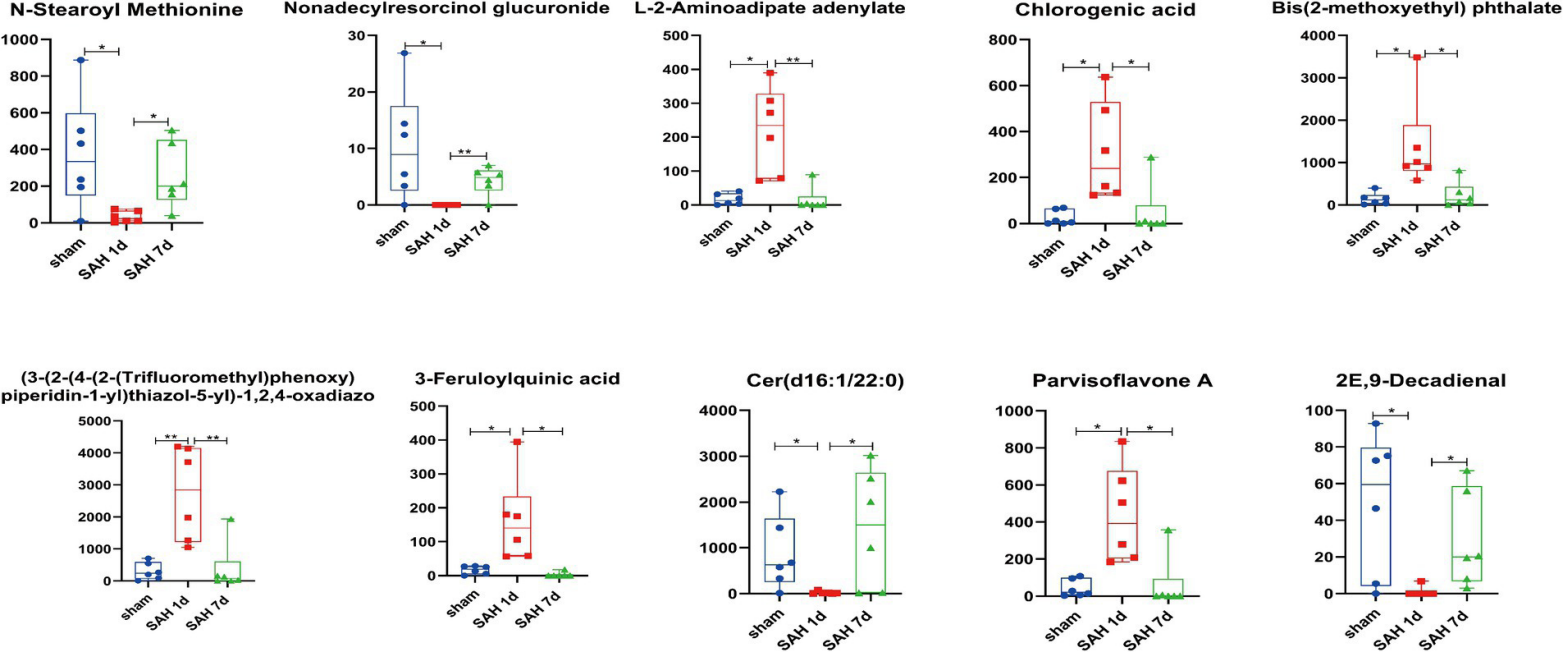
Block diagram of horizontal changes in differential metabolites (*p < 0.05, **p < 0.01, ***p < 0.001, *n* = 6).

Using the OPLS-DA model, differentially expressed metabolites (DEMs) are selected based on the combined criteria of *p* value <0.05 and VIP > 1. Compared with the sham surgery group, SAH found 540 DEMs on day 1; Compared with SAH 1d, SAH 7d discovered 254 DEMs, totaling 136 DEMs (Figure 1B; Supplementary Table S1). Utilizing metaboAnalyst5.0, we analyze 136 DEMs and their metabolic pathways (Figure 1C; Supplementary Table S2), discovering that these DEMs are enriched in pathways such as folate biosynthesis, the pentose phosphate pathway, arachidonic acid metabolism, sphingolipid metabolism, cysteine and methionine metabolism, pyruvate metabolism, arginine and proline metabolism and pyrimidine metabolism. Metabolites within these pathways may serve as potential biomarkers. Volcano plots of differentially expressed ions between diverse groups (sham group vs. SAH 1d group, SAH 1d group vs. SAH 7d group) are shown in Figures 1D, E respectively. Additionally, Figure 2 shows the expression of the 10 DEMs that may have the highest disease severity among the 136 DEMs.

### 3.2 Transcriptomic analysis

**Figure 3:**
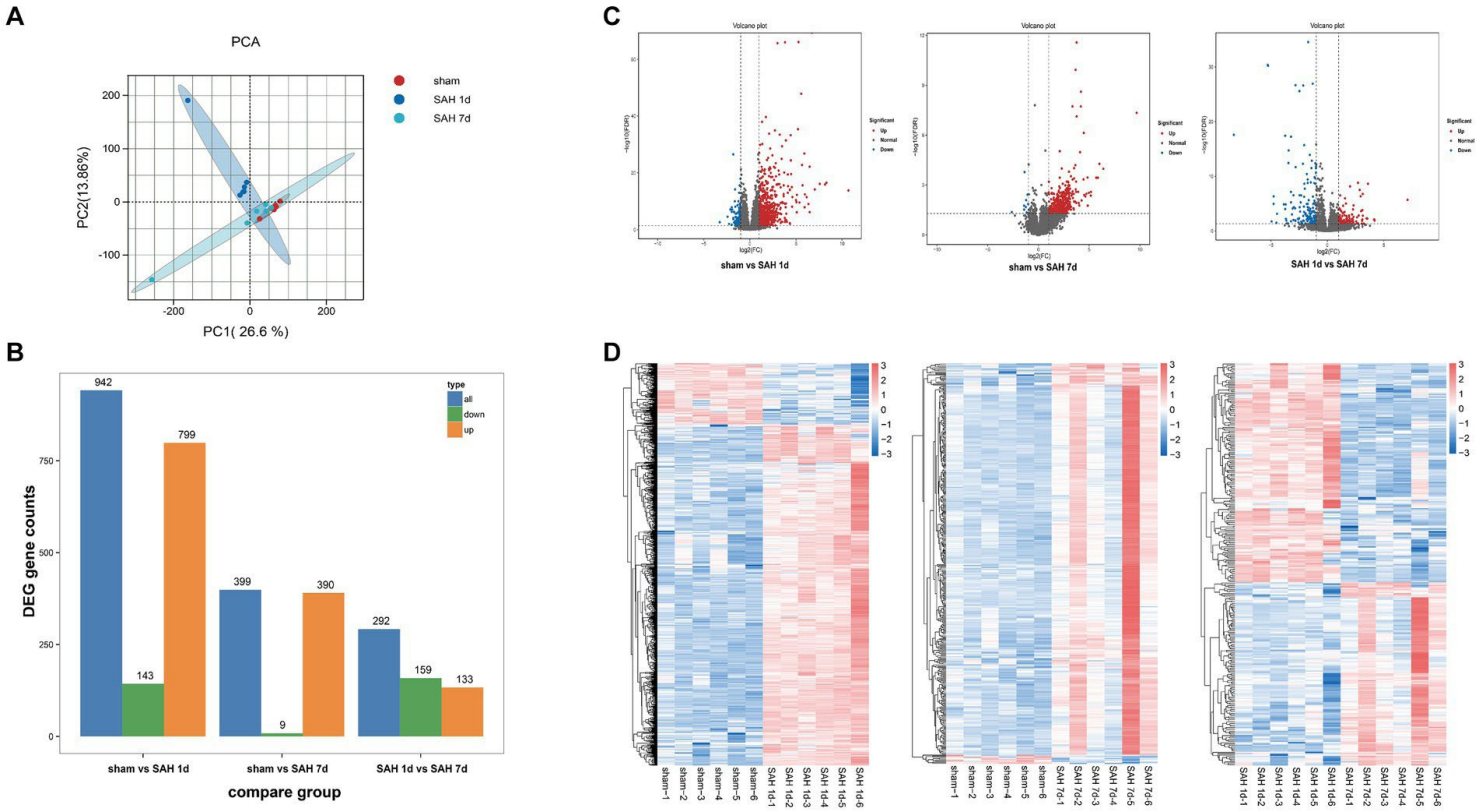
Transcriptomic analysis of differentially expressed genes. **(A)** Principal Component Analysis (PCA) evaluates the reproducibility of different product groups. **(B)** The number of upregulated and downregulated DEMs in different groups. **(C)** Volcano plots of differentially expressed genes in different groups, with red indicating upregulation and green indicating a downward adjustment. **(D)** A heatmap for clustering analysis of differentially expressed genes in different groups, with the red rectangle representing upregulated genes and the blue rectangle representing downregulated genes.

Before data analysis, we implemented a series of stringent quality control procedures, removing connector sequences and low-quality reads, resulting in approximately 2 million high-quality clean reads per sample. The proportions of quality scores Q20 and Q30 for each sample exceed 98 and 94%, respectively, with the GC content ranging between 47 to 49%, indicating the high quality of the data. The clean reads are aligned with the reference genome (Rattus_norvegicus.Rnor_6.0_release95.genome.fa), achieving an alignment efficiency ranging from 94.99 to 95.65%. This indicates a high utilization rate of the transcriptomic data (Supplementary Table S3). To compare gene expression levels across different samples, the FPKM (Fragments Per Kilobase of transcript per Million mapped reads) method is utilized. The results demonstrate that the distribution of gene expression levels is consistent across different samples (Supplementary Figures 3A,B). PCA is conducted on different samples, showing good separation between sample groups, indicating high intra-group correlation (Figure 3A). This is further confirmed through Spearman correlation analysis, validating good repeatability and reliability among different samples (Supplementary Figure 3C). The above results emphasize the robustness and consistency of the gene expression data, providing support for the reliability of the samples. A total of 1,198 differentially expressed genes (DEGs, |Fold Change| ≥ 2 and FDR < 0.05) are identified. Between the sham group and the SAH 1d group, 942 DEGs are identified, including 799 upregulated genes and 143 downregulated genes. Between the SAH 1d group and the SAH 7d group, 292 DEGs are identified, including 133 upregulated genes and 159 downregulated genes. Between the sham group and the SAH 7d group, 399 DEGs are identified, with both upregulated and downregulated genes totaling 390 and 9, respectively (Figure 3B). The overall distribution of DEGs is illustrated by volcano plots (Figure 3C). Hierarchical clustering analysis of the DEGs reveals distinct gene clusters and intergroup differences (Figure 3D).

To elucidate the specific biological functions of the DEGs between the sham group and the SAH 1d group, as well as between the SAH 1d group and the SAH 7d group, we performed Gene Ontology (GO) enrichment analysis. Through GO enrichment analysis, we gain a deeper understanding of the biological processes (BPs), cellular components (CCs), and molecular functions (MFs) that may be altered at different time points post-SAH.

**Figure 4:**
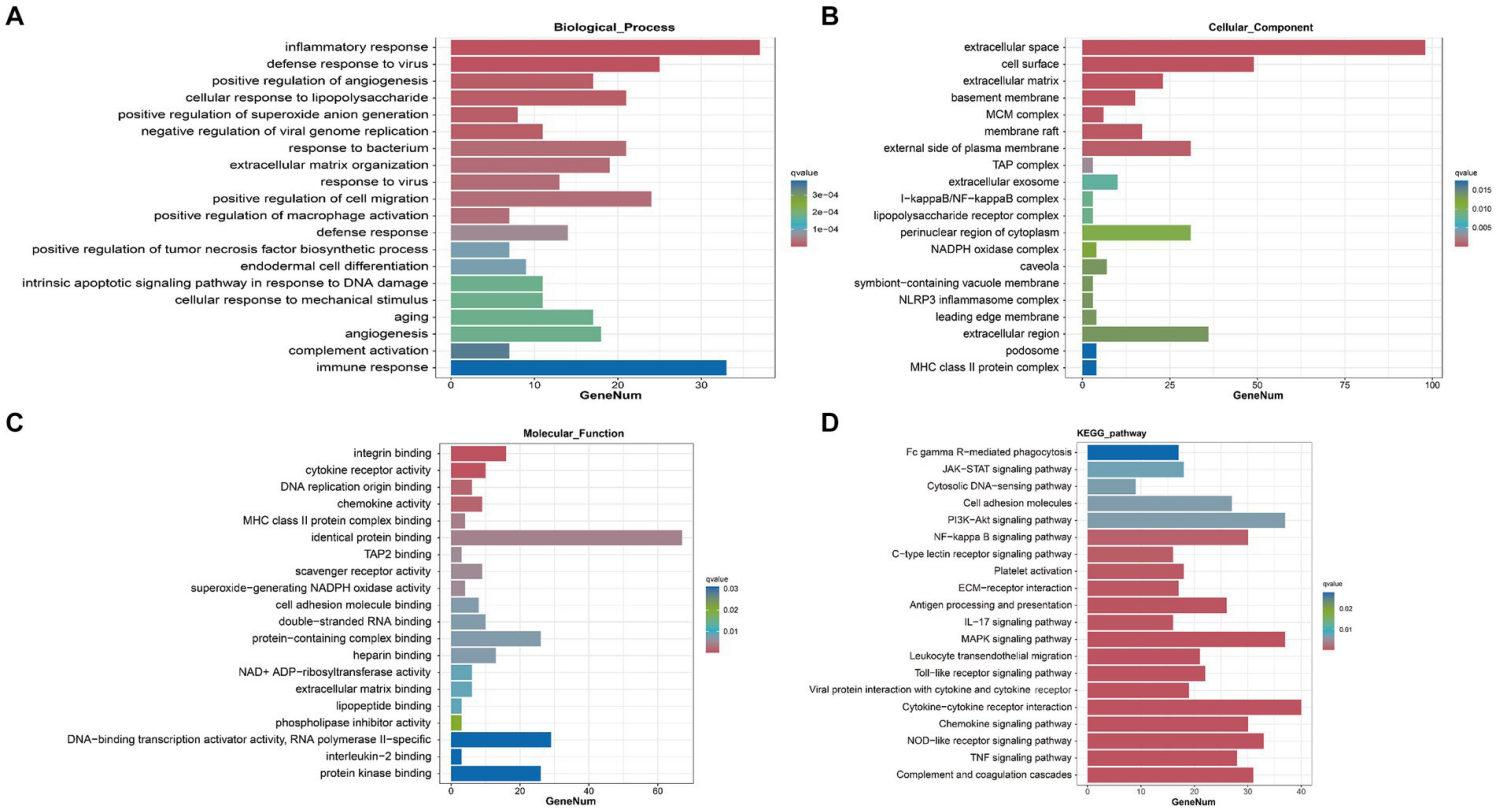
Transcriptomic analysis and joint analysis of the sham group and SAH 1d group. GO enrichment analysis of DEGs [BP **(A)**, CC **(B)**, MF **(C)**]. **(D)** KEGG enrichment analysis of DEGs.

In the SAH 1d group versus the Sham group, DEGs are enriched in various BPs, predominantly involving inflammatory and immune responses, regulation of angiogenesis, complement activation, cellular response to lipopolysaccharide, and defense response to viruses. In terms of CCs, the DEGs show enrichment in the extracellular space and matrix, MHC class II protein complex, MCM complex, and cell surface, which suggests their involvement in regulating these structural components. Regarding MFs, the DEGs are primarily implicated in pathways related to integrin binding, cytokine receptor activity, chemokine activity, and cell adhesion molecule binding (Figures 4A–C). Besides, in the SAH 7d group versus the SAH 1d group, DEGs are remarkably enriched in BPs related to the regulation of phagocytosis, engulfment, regulation of endothelial cell proliferation and cell adhesion, regulation of the inflammatory response, complement activation, and mediated synaptic pruning. These DEGs play a role in the negative regulation of inflammation and immunity, as well as positive regulation related to repair mechanisms. In terms of CCs, the DEGs are involved in pathways regulating the extracellular space, MHC class II protein complex, lysosome, collagen-containing extracellular matrix, and cell surface. For MFs, the DEGs are implicated in pathways modulating sulfiredoxin activity, cytokine activity, heme binding, and growth factor activity (Supplementary Figures S4A–C).

Further analysis of the DEGs is conducted through KEGG pathway analysis. The results reveal that, compared to the sham group, DEGs at SAH 1d are enriched in multiple inflammatory and immune pathways including Complement and coagulation cascades, Cytokine-cytokine receptor interaction, Chemokine signaling pathway, and the majority of these DEGs were upregulated (Figure 4D). This analysis underscores the significant activation of inflammation and immune response pathways shortly after SAH, highlighting the body’s immediate molecular response to injury and the potential targets for therapeutic intervention to modulate these responses. Compared to SAH 1d, the DEGs at SAH 7d are also dramatically enriched in inflammatory and immune pathways, containing Antigen processing and presentation, ECM-receptor interaction, and Th17 cell differentiation signaling pathways (Supplementary Figure 4D). However, the majority of these DEGs are downregulated. This shift in gene expression indicates a transition in the inflammatory and immune response from the acute phase following injury to a more controlled state, hinting at the body’s attempt to restore homeostasis and initiate repair processes.

### 3.3 Joint analysis of metabolism and transcription

**Figure 5:**
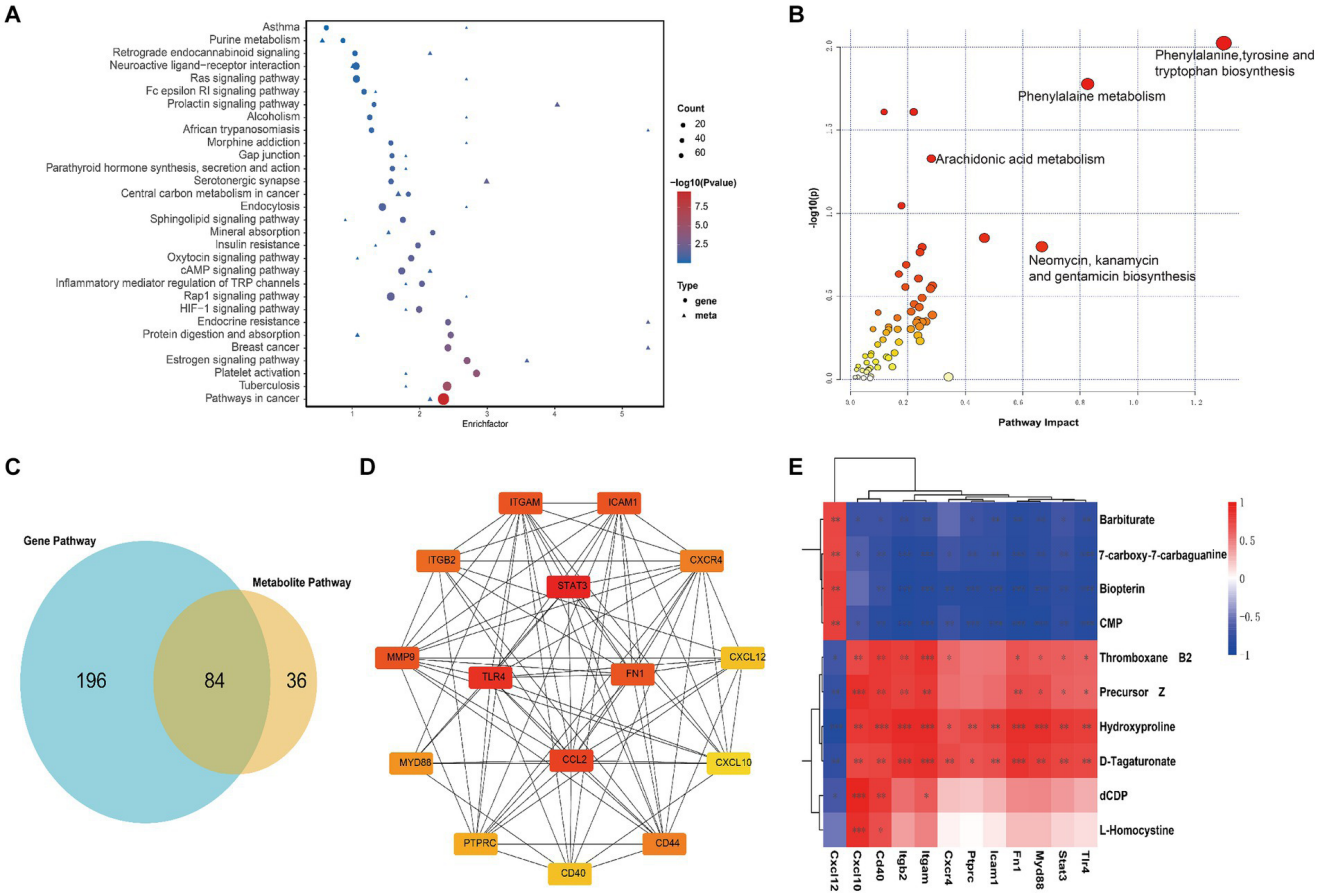
Joint analysis of transcriptomics and metabolomics. **(A)** Enrichment pathway analysis of differentially expressed genes and metabolites in KEGG, with circles representing the transcriptome and triangles representing the metabolome between Sham group and SAH 1d group. The size of the bubble represents the number of differential metabolites or genes, and the larger the number, the larger the dot. **(B)** Pathway enrichment analysis of differentially expressed genes and metabolites between Sham group and SAH 1d group. **(C)** Venn diagram of metabolic pathways enriched by metabolomics and transcriptomics between Sham group and SAH 1d group. **(D)** 15 core genes were selected from the protein interaction network of immune inflammation-related differentially expressed genes. **(E)** Correlation between core genes and specific metabolites (*p < 0.05, **p < 0.01, ***p < 0.001).

To further probe into the interrelationship between DEGs and DEMs, a joint analysis is conducted on the sham group and the SAH 1d group. The number of common pathways revealed by KEGG analysis is presented in Figure 5C, and some of the pathways prominently enriched with both DEGs and DEMs are displayed in Figure 5A. The results indicate that these common pathways are tightly linked to inflammation, immune and metabolism-related pathways. Further pathway enrichment analysis of DEGs and DEMs is conducted using the MetaboAnalyst5.0 platform, resulting in a metabolic pathway enrichment map (Figure 5B; Supplementary Table S4). The joint analysis results of the SAH 1d group and SAH 7d group are shown in Supplementary Figures S4E–G and Supplementary Table S5. Comparing these results with the enrichment outcomes between the SAH 1d group and the SAH 7d group reveals that both sets of data are closely associated with pathways such as Arginine and proline metabolism, Arachidonic acid metabolism, Folate biosynthesis, Sphingolipid metabolism, Cysteine, and methionine metabolism, Pyrimidine metabolism and Inositol phosphate metabolism, which are consistent with those identified in Figure 1C.

**Figure 6:**
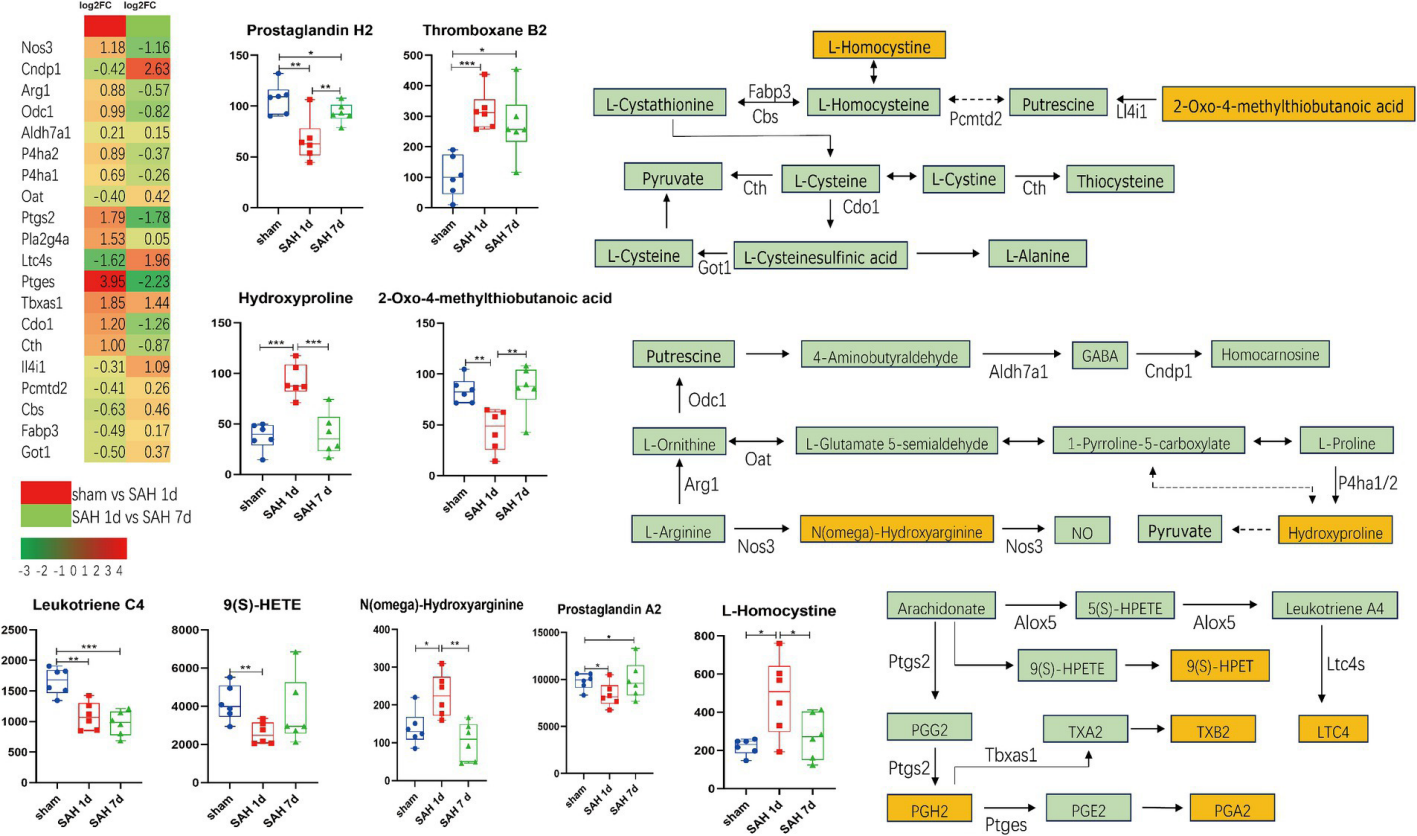
Simplified diagram of arginine and proline metabolism, arachidonic acid metabolism, and arachidonic acid metabolism pathways, with red indicating gene upregulation and green indicating gene downregulation (*p < 0.05, **p < 0.01, ***p < 0.001, *n* = 6).

**Figure 7:**
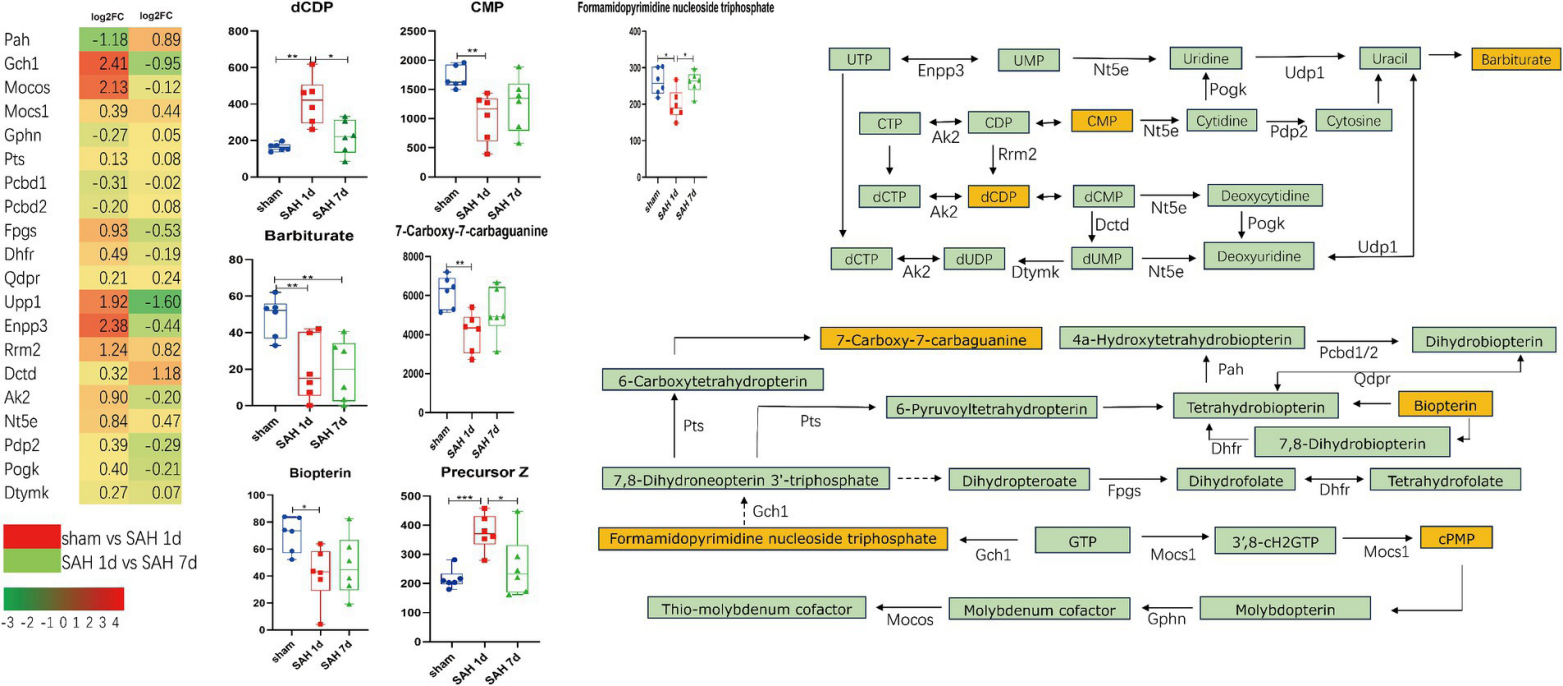
Simplified diagram of folate synthesis and pyrimidine metabolism pathways, with red indicating gene upregulation and green indicating gene downregulation (*p < 0.05, **p < 0.01, ***p < 0.001, *n* = 6).

The metabolites and their related genes within these enriched pathways above are detailed in Table 1, followed by representative metabolic pathway diagrams (Figures 6, 7). In the comparison between the sham group and the SAH 1d group, metabolites such as Hydroxyproline, N(omega)-Hydroxyarginine, Thromboxane B2, Precursor Z, L-Homocystine, and dCDP show increased expression. In contrast, Prostaglandin A2, Leukotriene C4, Prostaglandin H2, 9(S)-HETE, Formamidopyrimidine nucleoside triphosphate, Barbiturate, Biopterin, 7-carboxy-7-carbaguanine, 2-Oxo-4-methylthiobutanoic acid, and CMP experience a decrease in expression levels. However, when comparing the SAH 1d group with the SAH 7d group, the expression levels of these metabolites are opposite (Figures 6, 7).

**Table 1:** Differentially expressed genes and metabolites in different pathways.

Pathway	Gene	Metabolite
Arginine and proline metabolism	Nos3; Cndp1; Arg1; Odc1; Aldh7a1; P4ha2; P4ha1; Oat	Hydroxyproline; N(omega)-Hydroxyarginine
Arachidonic acid metabolism	Ptgs2; Pla2g4a; Ltc4s; Ptges; Tbxas1	Thromboxane B2; Prostaglandin A2; Leukotriene C4; Prostaglandin H2; 9(S)-HETE
Folate biosynthesis	Pah; Gch1; Mocos; Mocs1; Pts; Pcbd1; Pcbd2; Fpgs; Dhfr; Qdpr	Formamidopyrimidine nucleoside triphosphate; Precursor Z; Biopterin; 7-Carboxy-7-carbaguanine
Pyrimidine metabolism	Upp1; Enpp3; Rrm2; Dctd; Ak2; Nt5e; Pdp2; Pogk; Dtymk	Barbiturate; CMP; dCDP
Cysteine and methionine metabolism	Cdo1; Cth; Ll4i1; Pcmtd2; Cbs; Fabp3; Got1	2-Oxo-4-methylthiobutanoic acid; L-Homocystine
Inositol phosphate metabolism	Itpkb; Itpkc; Plce1; Plcd1; Plcb2	D-Tagaturonate
Sphingolipid metabolism	Acer2; Sphk1	Sphinganine

To further explore the potential connections between metabolites and immune-inflammatory genes, this study particularly focuses on comparisons between the sham group and the SAH 1d group. DEGs related to immune-inflammatory are extracted from the KEGG database and combined with DEMs associated with the above pathways. Spearman correlation network analysis is conducted (Supplementary Figure S5; Supplementary Table S6) and the results were visualized using the Cytoscape tool (Supplementary Figure S6). The above analysis reveals significant correlations between these DEGs and DEMs. Further, using protein–protein interaction (PPI) networks and the CytoHubba plugin, top 15 hub genes associated with immune inflammation are identified, including STAT3, TLR4, CCL2, MMP9, FN1, ITGAM, ICAM1, ITGB2, CD44, CXCR4, MYD88, PTPRC, CXCL12, CD40 and CXCL10 (Figure 5D). Figure 5E shows further correlation analysis between these hub genes and specific DEMs. These findings further affirm the close connection between immune-inflammatory genes and specific DEMs, providing a wealth of information that transcends single-omics studies.

## 4 Discussion

Subarachnoid hemorrhage (SAH) is the third most common type of stroke, typically caused by the rupture of intracranial aneurysms. It is an acute and highly fatal hemorrhagic stroke. The initial bleeding event can lead to increased intracranial pressure, dysregulation of cerebral perfusion pressure, impaired microvascular function, delayed cerebral ischemia, and extensive systemic dysfunctions (Claassen and Park, 2022; Lauzier and Athiraman, 2024). In the pathogenesis of SAH, the activation of brain immune-inflammatory cells and the release of inflammatory factors play a pivotal role in damaging the blood–brain barrier (BBB) and causing neuronal injury, thereby exacerbating brain damage. In the present study, the results from KEGG and GO analyses indicate that DEGs within immune and inflammatory pathways are broadly upregulated. We have screened core genes related to immunity and found that these genes are strongly associated with oxidative stress, endothelial cell injury, and disruption of the BBB. Similarly, changes in metabolites are crucial for the pathological mechanisms of SAH, but while current understanding remains limited. Our study reveals a close connection between immune-inflammatory-related DEGs and specific DEMs. This underscores the complex interplay between genomic and metabolomic alterations in SAH, highlighting the importance of integrating multiple omics approaches to fully understand the pathophysiology of SAH and identify potential therapeutic targets. Our preliminary findings demonstrate that the pathogenesis of SAH may be associated with alterations in metabolic pathways related to arachidonic acid metabolism, cysteine and methionine metabolism, arginine and proline metabolism, folate biosynthesis, and purine metabolism. Further analysis reveals that metabolites in these metabolic pathways are closely related to oxidative stress, immune inflammation, endothelial cell damage, and BBB breakdown.

Arachidonic acid and its derivatives are crucial substances involved in oxidative stress and inflammation, capable of inducing the production of inflammatory factors by cells, thus playing a vital role in the immune-inflammatory process. Prostaglandin H2 (PGH2) is synthesized from arachidonic acid under the influence of COX2, which is encoded by the PTGS2. In mouse models deficient in the PTGS2 gene, cerebral ischemia leads to increased sensitivity to neurotoxicity, enhanced oxidative stress, and exacerbation of neuronal death (Iadecola et al., 2001). PGH2 can be converted into a variety of biologically active compounds, including PGE2, PGD2, PGA2, TXA2, TXB2, and LTB4. Inhibiting the production of prostaglandins can help maintain endothelial cell calcium ion balance, reduce inflammation and excitotoxicity, and prevent apoptosis in endothelial cells and neurons (Yagami et al., 2016; Xu et al., 2017). Meanwhile, an increase in TXA2 and TXB2 contributes to promoting platelet aggregation, blood coagulation, and vasoconstriction, which can alleviate the destruction of the BBB. These effects collectively mitigate brain injury. Additionally, the conversion products of PGH2 can regulate the excitability of synapses in the hippocampus and increase synaptic plasticity by binding to specific receptors, thereby improving long-term behavioral deficits (Heneka et al., 2015; Yagami et al., 2016; Wood et al., 2019). In SAH, leukotriene B4 (LTB4) promotes neuronal death in the cerebral cortex and augments brain edema by increasing inflammation and oxidative stress enhancing microvascular permeability, inducing leukocyte chemotaxis, elevating myeloperoxidase levels (Dvash et al., 2015; Ye et al., 2018). 9-Hydroperoxyeicosatetraenoic acid (9-HPETE) is a fatty acid derivative formed from arachidonic acid through the action of lipoxygenases, which can be converted into biologically active 9-HETE. 9-HPETE and its metabolites play roles in various biological processes, including inflammation, immune responses, cell proliferation, and apoptosis. Middle-chain HETEs (including 9-HETE) significantly stimulate the phosphorylation of ER1/2 and induce the activation of the NF-κB pathway, contributing to oxidative stress and pro-inflammatory effects and higher levels of 9-HETE are associated with an increased incidence of acute myocardial infarction in patients, as well as positive correlations with certain inflammatory and cardiac biomarkers, such as TNFα (Horn et al., 2013; Maayah et al., 2017; Morris et al., 2019; Huang et al., 2020). We speculate that arachidonic acid and its derivatives are involved in the pathological processes of SAH and secondary brain injury.

Arginine, proline, and cysteine metabolism are crucial in regulating brain function and pathological processes. N(ω)-hydroxyarginine (NOHA) is converted from arginine under the action of nitric oxide synthase (NOS), further generating NO; besides, it is also recognized as an inhibitor of NOS, showing high affinity for human arginase I. By modulating arginase activity, NOHA promotes the hydrolysis of L-arginine into L-ornithine and urea, thereby indirectly affecting the production of nitric oxide (Di Costanzo et al., 2010). After SAH, the fluctuation of NO levels in the brain is closely associated with a series of pathological responses, including early vascular constriction, platelet aggregation, vascular endothelial cell injury, destruction of the BBB, and later events like delayed cerebral vasospasm and neuronal and axonal damage (Sehba and Bederson, 2011; Osgood, 2021). However, NO also regulates the nuclear factor NF-κB, which not only promotes ferroptosis in M1-type microglia but also drives the polarization shift of microglia from the M1 to the M2 phenotype, thereby alleviating neuroinflammation (Qu et al., 2022; Tian et al., 2022). We observe that 1 day after SAH, the activation of Arg1 and Odc1, and the inhibition of Cndp1 and OAT, might lead to an increase in GABA expression. 7 days after SAH, the reverse expression of these genes could potentially cause a decrease in GABA expression. In SAH, GABA can participate in inhibiting pyroptosis and the expression and release of inflammatory factors (such as NLRP3, ASC, caspase-1) (Shi et al., 2022). Taurine, by activating GABA receptors, can reduce lipid peroxidation and alleviate post-SAH brain edema, oxidative stress, and BBB disruption (Liu et al., 2022). The decrease in GABA levels in the motor cortex following SAH may lead to impaired cortical neuron function, resulting in long-term neurological deficits (Wang et al., 2020; Sugita et al., 2023). 2-Oxo-4-methylthiobutanoic acid (MTOB) is both an intermediate and end product in the methionine salvage pathway, serving as a precursor for the synthesis of methionine, cysteine, and other important sulfur-containing compounds. MTOB can reduce the activity of ornithine decarboxylase (Tang et al., 2006). Homocysteine has potential neurovascular toxicity through its oxidative effects, activation of tissue factors, and generation of free radicals leading to damage in histone acetylation, dysfunction of the activated ubiquitin system, formation of microthrombi, and a decrease in nitric oxide (NO) levels (Dardik et al., 2000; Wan et al., 2022). Homocysteine induces oxidative stress and upregulates MMPs, leading to decreased expression of tight junction proteins, which results in increased permeability of the BBB. In addition, homocysteine can activate the acid sphingomyelinase-ceramide pathway, inducing apoptosis in cerebral endothelial cells (Lee et al., 2013; Kalani et al., 2014). After SAH, an increase in homocysteine levels in the serum may make it a potential molecular predictor of delayed cerebral ischemia (Liu et al., 2020; Zhang et al., 2021). The changes in the above metabolites may be involved in oxidative stress and blood–brain barrier disruption after SAH.

Folic acid and its derivatives are nutrients essential for various cellular functions, playing a key role in maintaining the activity of enzymes such as phenylalanine hydroxylase, nitric oxide synthase, alkylglycerol monooxygenase, and enzymes related to neurotransmitter synthesis (Werner et al., 2011). GCH1 is the rate-limiting enzyme in the synthesis of tetrahydrobiopterin (BH4), also regulating NO signal transduction, inhibiting ferroptosis, thereby mitigating endothelial damage and suppressing oxidative stress (Kraft et al., 2020; Gupta and Kumar, 2023; Xiao et al., 2023). The alternative pathway involving DHFR for BH4 synthesis, along with an increase in QDPR and PAH expression, can promote the production of BH4. BH4 exhibits strong scavenging abilities against superoxide anion radicals, significantly reducing radical formation and endothelial cell death caused by NO; however, low concentrations of BH4 can exacerbate NOS uncoupling, leading to the production of superoxide and peroxynitrite, enhancing oxidative stress and worsening microvascular damage. Notably, the oxidation of BH4 may lead to an increase in oxygen-derived free radicals, closely associated with vascular endothelial dysfunction (Shimizu et al., 1998; Thöny et al., 2008; Werner et al., 2011). In mice infected with the rabies virus, BH4 can mitigate the damage to the central nervous system by modulating the immune-inflammatory response (Brito et al., 2021). Tetrahydrofolate (THF), the active form of folic acid, can alleviate oxidative stress and enhance the repair capabilities of neural cells through the PTEN/Akt/mTOR signaling pathway (Zhang et al., 2022). Moreover, high levels of THF in the cerebrospinal fluid can promote the proliferation of arachnoid cells, and enhance the function of the arachnoid layer, thereby alleviating hydrocephalus (Jimenez et al., 2019). Pyrimidine metabolism regulates cell signaling and energy metabolism, helping to maintain cellular homeostasis. Barbiturate, a metabolite of uracil, has been used for a long time as an anticonvulsant and hypnotic acting on GABA receptors (Löscher and Rogawski, 2012). In SAH, barbiturates can induce metabolic suppression, thereby reducing cerebral blood flow and brain blood volume, which in turn lowers intracranial pressure and improves prognosis. High doses of barbiturates can also reduce inflammatory response caused by SAH, lowering levels of IL-6, IL-1β, and white blood cell counts in the cerebrospinal fluid (Muroi et al., 2008). UPP1 is a key enzyme in pyrimidine metabolism, capable of activating the AKT signaling pathway, thereby regulating downstream proteins that promote cell proliferation, and migration, and inhibit apoptosis, thus reducing DNA damage and promoting angiogenesis (Du et al., 2024); additionally, apart from its role in the formation of UMP, ENPP3 also reduces ATP concentrations, diminishing the activity of basophils and mast cells, thus playing a role in immune-inflammatory responses (Stone et al., 2010). In Figure 7, pyrimidine-related metabolites are closely associated with immune-inflammatory genes. Barbiturate and CMP show a negative correlation with the vast majority of immune-inflammatory related genes, especially hub genes; whereas dCDP shows the opposite effect. However, in SAH, the above-mentioned metabolites have been rarely studied and are worth further exploration.

## 5 Conclusion

We employed RNA-seq and metabolomics techniques to deeply analyze the changes in gene expression patterns and metabolite abundances at diverse time points in the SAH model.

Through this study, we have identified several metabolic pathways related to inflammatory immune response and endothelial cell damage, which provides a significant theoretical basis for further exploring the potential molecular pathological features of SAH and new insights for the diagnosis, treatment, and prognosis monitoring of SAH.

## Data Availability

The data generated for this study can be found in the Sequence Read Archive, https://www.ncbi.nlm.nih.gov/geo/query/acc.cgi?acc=GSE273799. Data will be made publically available on August 2nd, 2025. Prior to this date the data will be available upon request from the authors.

## Footnotes

^1^ http://www.biocloud.net/

^2^ https://www.omicshare.com/tools/
