# Correction: Metabolome and transcriptome integration reveals cerebral cortical metabolic profiles in rats with subarachnoid hemorrhage

**DOI:** 10.3389/fnagi.2026.1834508

**Published:** 2026-04-28

**Authors:** Haoran Lu, Teng Xie, Shanshan Wei, Yanhua Wang, Huibing Li, Baochang Luo, Xiaohong Qin, Xizhi Liu, Zilong Zhao, Zhibiao Chen, Rui Ding

**Affiliations:** 1 Department of Neurosurgery, Renmin Hospital of Wuhan University, Wuhan, China; 2 Department of Neurosurgery, Hanchuan Renmin Hospital, Hanchuan, China; 3 Department of Oncology, Wuchang Hospital Affiliated to Wuhan University of Science and Technology, Wuhan, China

**Keywords:** subarachnoid hemorrhage, transcriptome, metabolome, secondary brain injury, inflammation

The supplemental file “**Supplementary Data Sheet 1**” was erroneously omitted from the published article. This file should have included **Supplementary Figures 1–6**. The file(s) have now been published alongside the article as intended.

The original version of this article has been updated.

