# Supplementary material for: Metabolome and transcriptome integration reveals cerebral cortical metabolic profiles in rats with subarachnoid hemorrhage
Source: Front Aging Neurosci. 2024 Aug 21;16:1424312. doi: 10.3389/fnagi.2024.1424312 (PMC11371592; doi:10.3389/fnagi.2024.1424312)
Supplement: Supplementary file 10 [file Data_Sheet_1.DOC]

**Supporting Information**

**Metabolome and transcriptome integration reveals cerebral cortical metabolic profiles in rats with subarachnoid hemorrhage**

Haoran Lu^a,#^, Teng Xie^b,#^, Shanshan Wei^c,#^, Yanhua Wang^b^, Huibing Li^b^, Baochang Luo^b^, Xiaohong Qin^a^, Xizhi Liu^a^, Zilong Zhao^a^, Zhibiao Chen^a^,*, Rui Ding^a^,*

^a^Department of Neurosurgery, Renmin Hospital of Wuhan University, Wuhan, 430060, China.

^b^Department of Neurosurgery, Hanchuan Renmin Hospital, Hanchuan, Hubei 431600, China.

^c^Department of Oncology, Wuchang Hospital Affiliated to Wuhan University of Science and Technology, Wuhan, 430063, China.

^#^These authors (Haoran Lu, Teng Xie and Shanshan Wei) contributed equally.

*Correspondence to: Department of Neurosurgery, Renmin Hospital of Wuhan University, 99 Zhang Zhidong Street, Wuhan, 430060, China. E-mail addresses: [chzbiao@126.com](mailto:chzbiao@126.com) (Zhibiao Chen, co-corresponding author), [rui-ding@hotmail.com](mailto:rui-ding@hotmail.com) (Rui Ding, corresponding author).

**Supplementary Figure 1**


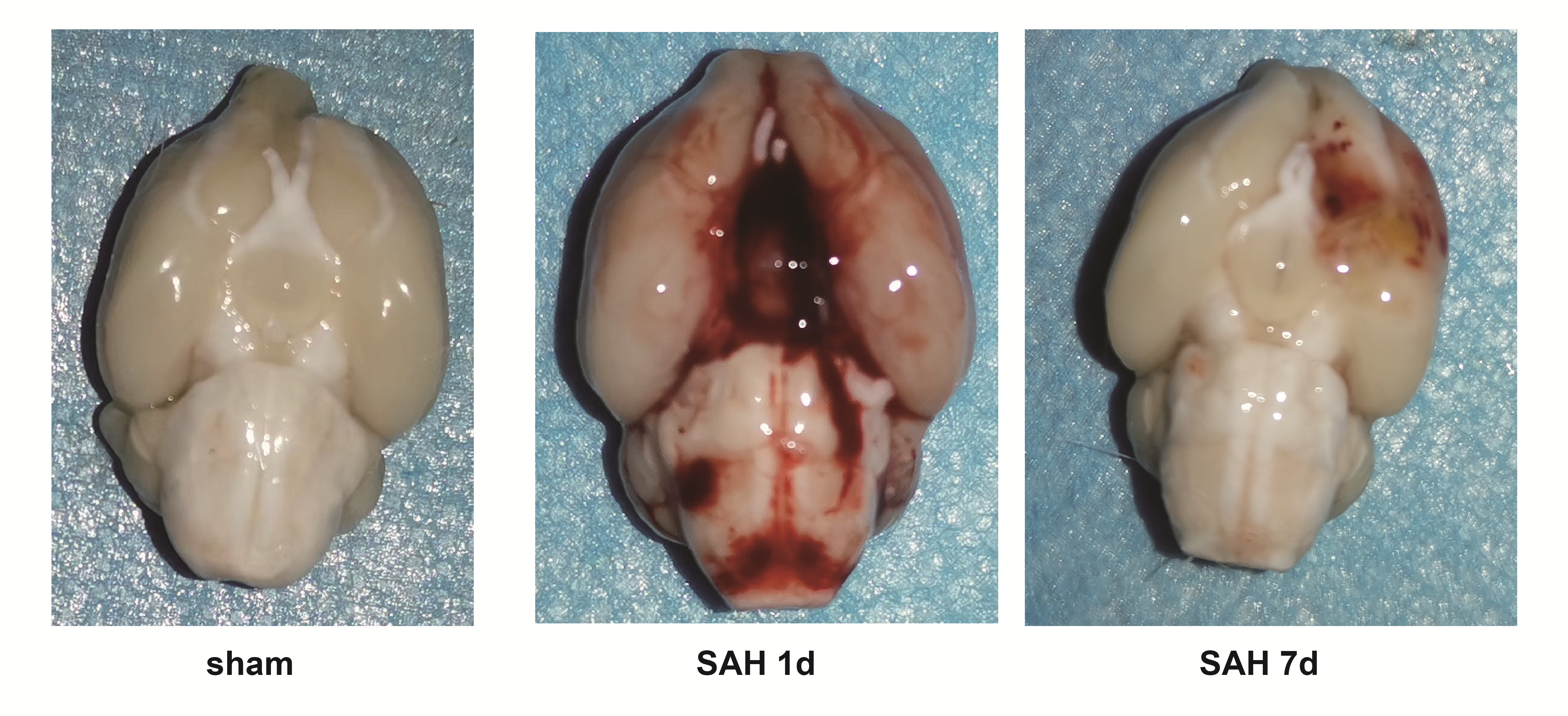


**Pictures of typical rat subarachnoid hemorrhage in different groups**

**Supplementary Figure 2**


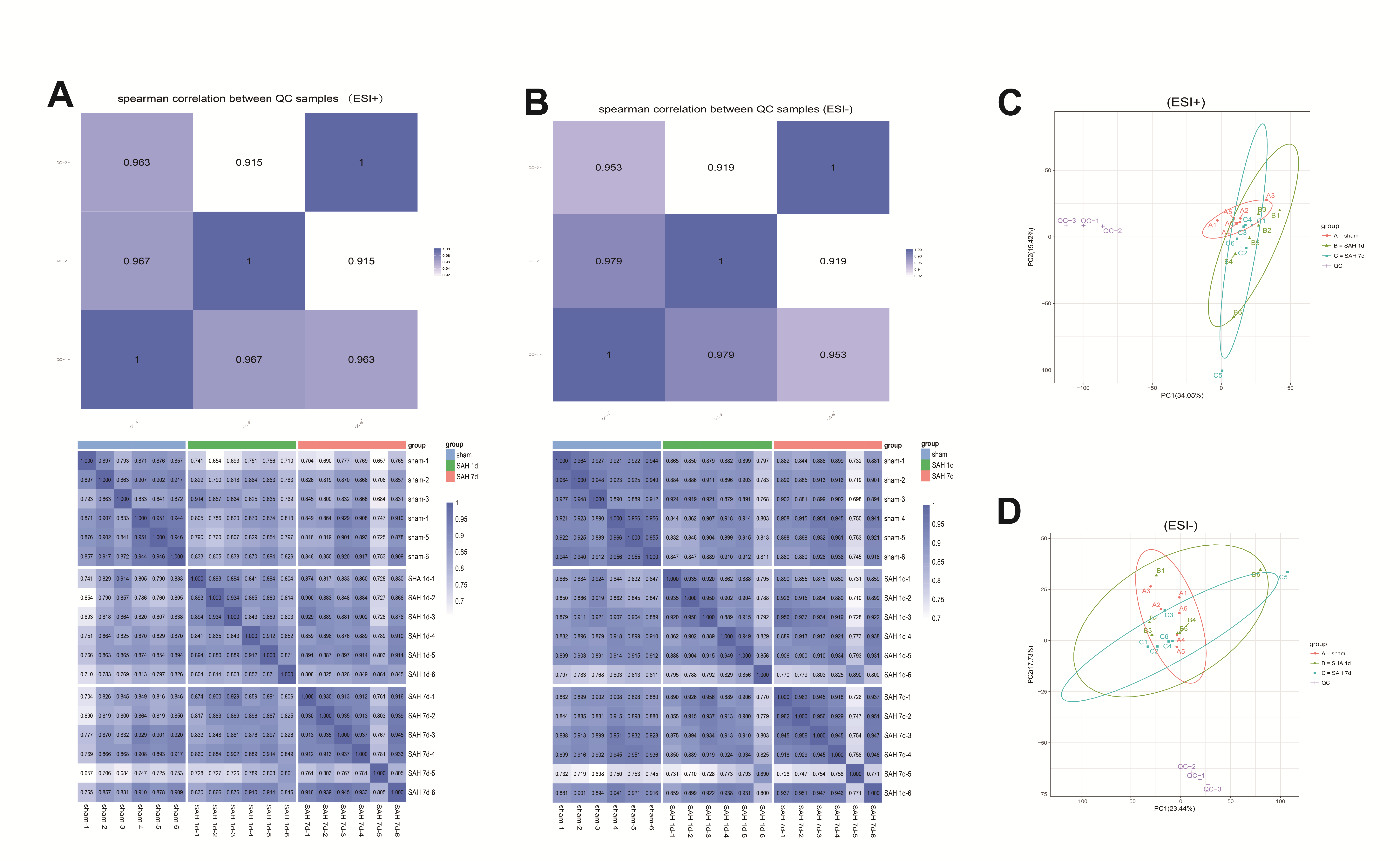


**Correlation between different samples and groups**

(A) Correlation between different groups of samples in positive ion mode. (B) Correlation between different groups of samples in negative ion mode. Under positive (C) and negative (D) ion modes, principal component analysis (PCA) evaluates the repeatability of different product groups. Group A is the sham group, and Group B is the SAH 1d group; Group C is SAH 7d group.

**Supplementary Figure 3**


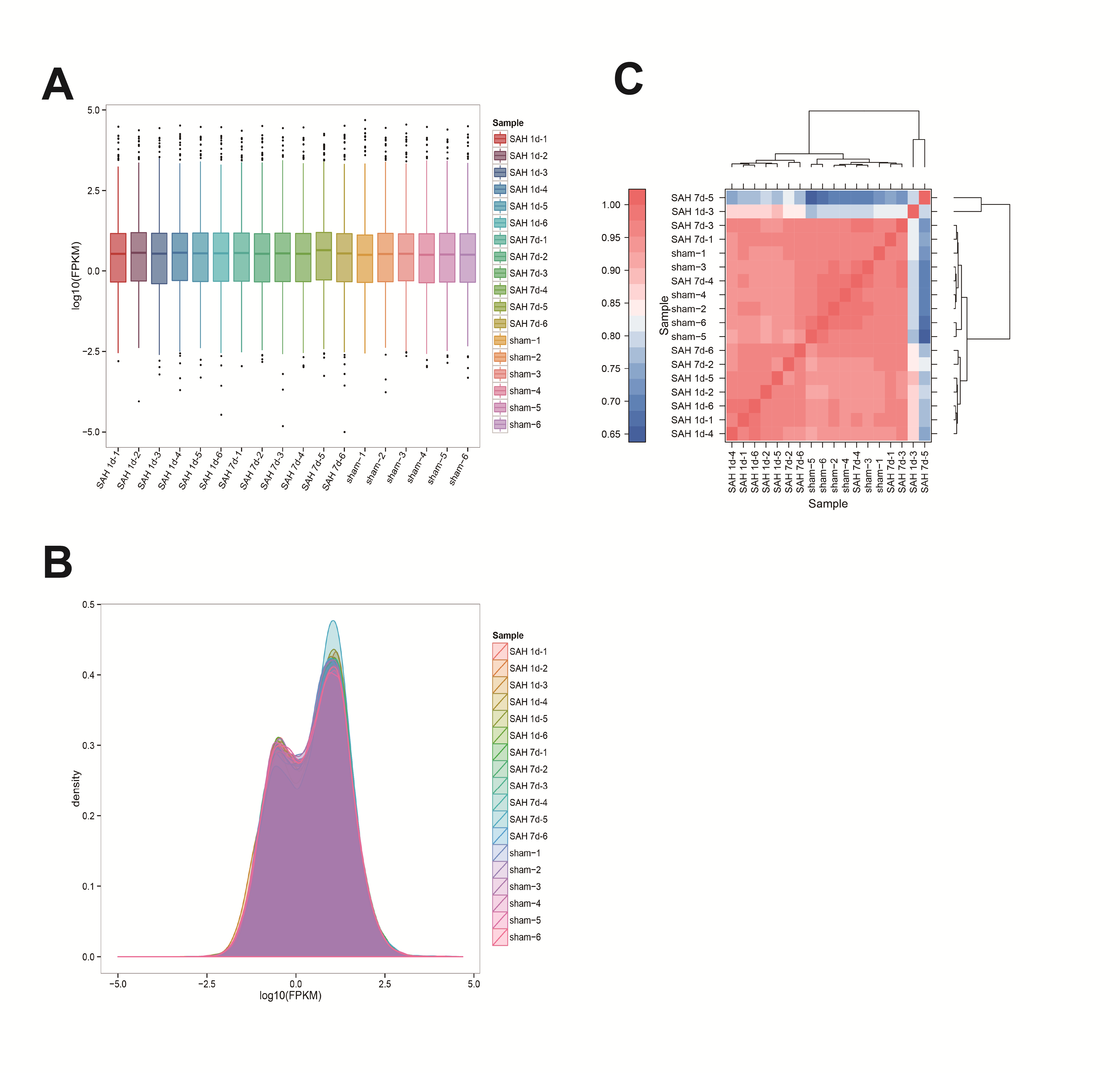


**Correlation between different samples and groups**

Estimate the distribution of gene expression levels in different sample groups using boxplot (A) and density distribution comparison plot (B). FPKM, the number of fragments per thousand kilograms of base read per million mappings. (C) Spearman correlation analysis of gene expression profiles from different samples.

**Supplementary Figure 4**


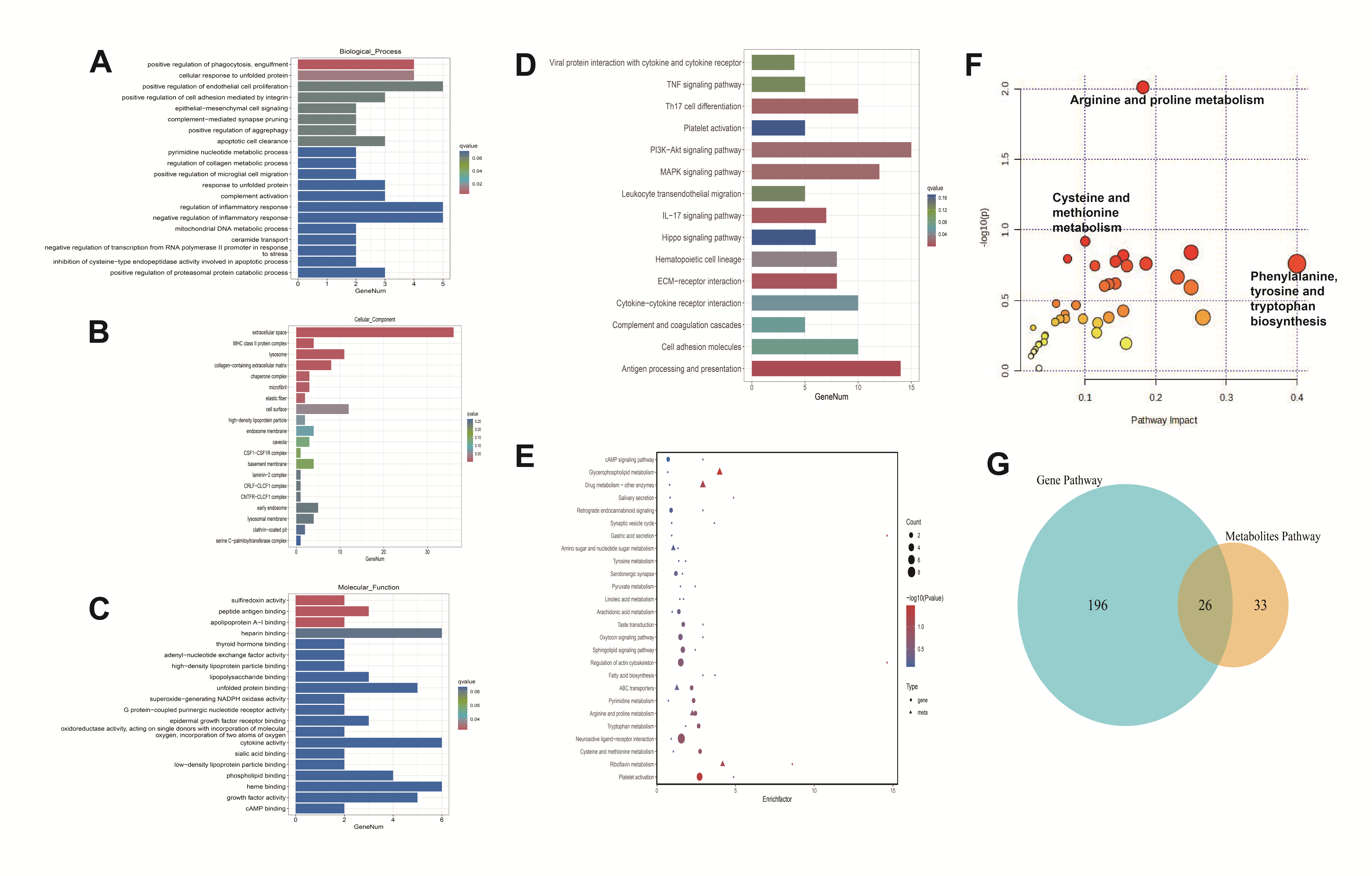


**Transcriptomic analysis and joint analysis of SAH 1d and SAH 7d.**

GO enrichment analysis of DEGs (BP (A), CC (B), MF (C)). (D) KEGG enrichment analysis of DEGs (E) Venn diagram of metabolic pathways enriched by metabolomics and transcriptomics (F) pathway enrichment analysis of differentially expressed genes and metabolites (G) enrichment analysis of differentially expressed genes and metabolites in KEGG, with circles representing the transcriptome and triangles representing the metabolome; The size of the bubble represents the number of differential metabolites or genes, and the larger the number, the larger the dot.

**Supplementary Figure 5**


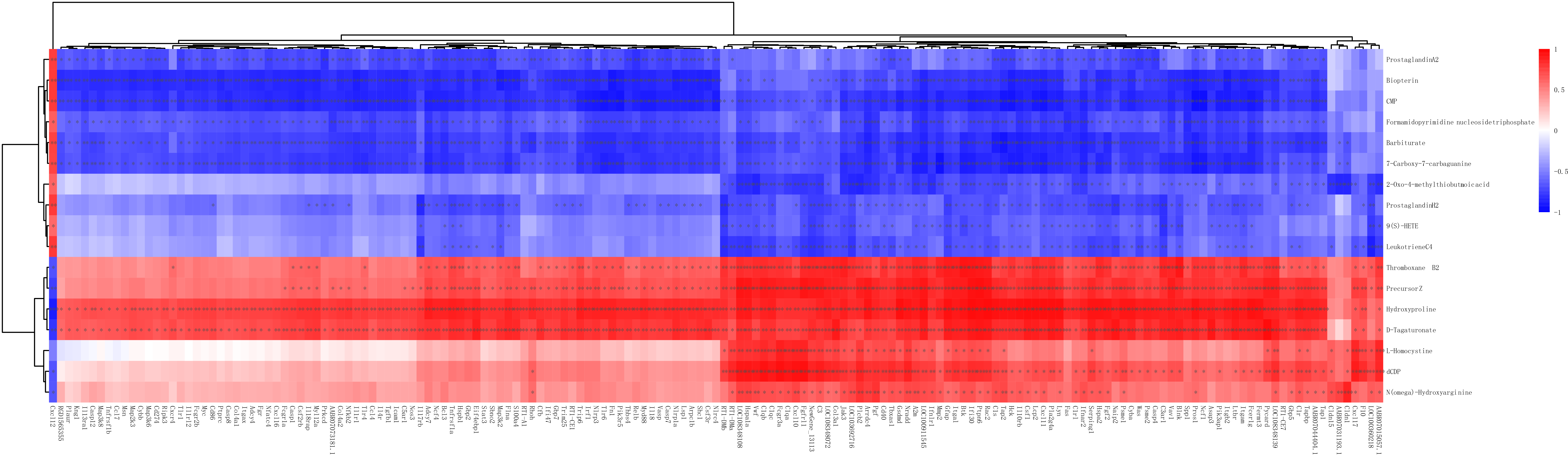


**Heat map of correlation between immune inflammation-related DEGs and specific DEMs**

Differential genes and metabolites were screened according to correlation coefficient (CC) and correlation p-value (CCP), screening threshold: | CC |>0.80 and CCP<0.05. (*p<0.05, **p<0.01, ***p<0.001, n=6)

**Supplementary Figure 6**


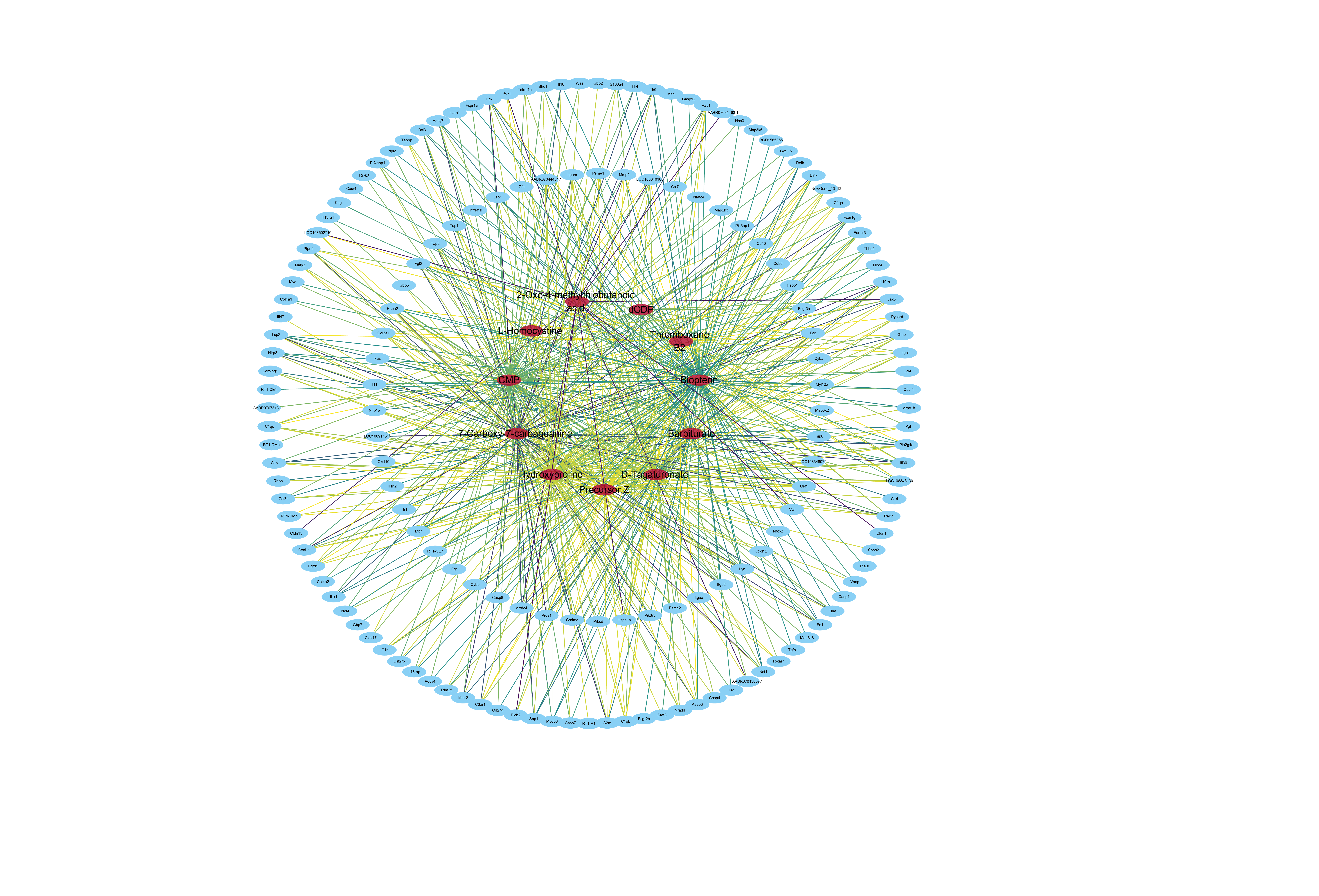


**Interaction map between DEGs related to immune inflammation and specificDEMs**
